# The safe insertion of peripheral intravenous catheters: a mixed methods descriptive study of the availability of the equipment needed

**DOI:** 10.1186/2047-2994-1-15

**Published:** 2012-04-20

**Authors:** Bryony Dean Franklin, Vashist Deelchand, Matthew Cooke, Alison Holmes, Charles Vincent

**Affiliations:** 1 Imperial Centre for Patient Safety and Service Quality, Imperial College, London, UK; 2 Warwick Medical School, University of Warwick, Coventry, UK; 3 Imperial College Healthcare NHS Trust, London, UK; 4 Centre for Infection Prevention and Management, Imperial College, London, UK; 5 UCL School of Pharmacy, London, UK

**Keywords:** Cannulation, Patient safety, Equipment, Care bundles, Hospital acquired bacteraemia, Systems reliability

## Abstract

**Background:**

Intravenous cannulation is undertaken in a high proportion of hospitalised patients. Much international attention has been given to the use of care bundles to reduce the incidence of infection in these patients. However, less attention has been given to the systems required to ensure availability of the equipment needed to support these care bundles. Our objectives were to assess how reliably the equipment recommended for a peripheral intravenous care bundle was available for use, and to explore factors which contributed to its non-availability.

**Methods:**

We studied 350 peripheral cannula insertions in three NHS hospital organisations across the UK. Staff inserting cannulae were asked to report details of all equipment problems. Key staff were then interviewed to identify the causes of problems with equipment availability, using semi-structured qualitative interviews and a standard coding frame.

**Results:**

47 equipment problems were recorded during 46 of 350 cannulations, corresponding to a reliability of 87%, or 94% if problems with sharps disposal were excluded. Overall reliability was similar in all three organisations, but the types of problem varied. Interviews revealed a variety of causes including issues associated with purchasing policies, storage facilities, and lack of teamwork and communication in relation to reordering. The many human factors related to the supply chain were highlighted. Often staff had adopted work-arounds to deal with these problems.

**Conclusions:**

Overall, 87% of cannulations had the correct and functional equipment available. Different problems were identified in different organisations, suggesting that each had resolved some issues. Supply chain management principles may be useful to support best practice in care bundle delivery.

## Background

Insertion of peripheral intravenous catheters is one of the most common invasive procedures performed in hospitals. It is estimated that 200 million peripheral intravenous catheters are used annually in the US [1], while according to the Scottish National Prevalence survey, one in three UK inpatients have at least one peripheral venous catheter *in situ*[2]. Intravenous cannulation has the potential to introduce infection into the local tissues at the site of cannulation or directly into the blood stream. The incidence of local or bloodstream infections associated with peripheral intravenous catheters is usually low; however, due to the high frequency with which peripheral catheters are used, serious infectious complications produce considerable annual morbidity [3]. To reduce the incidence of patient harm during intravenous cannulation, improvements in the reliability of the process of delivery of care have been proposed. The US Center for Disease Control has produced extensive evidence-based guidelines for the prevention of infection associated with peripheral intravenous cannulae and central venous catheters [3]. In England, the Department of Health devised the ‘Saving Lives’ programme consisting of High Impact Interventions (“care bundles”) [4] to promote compliance with essential elements in care delivery and reduce variability of practice. Care bundles consist of key clinical procedures or care elements which, when performed together, help to reduce the risk of infection [5]. However, the availability of the right equipment when needed is also critical to ensure that frontline staff perform their tasks consistently, thus achieving the aim of the care bundle. The reliability of routine processes, the outcome of which is not catastrophic to patients, is often overlooked in healthcare [6]. It is argued that in order to achieve high reliability in healthcare, reliability of these routine processes needs improvement [6]. Studies published to date have focused on central venous catheter insertion, and have highlighted the equipment problems frontline staff may encounter. For instance, a 2004 study found that, to comply with existing guidelines for central venous catheter insertion, a physician went to eight different places to collect the equipment needed for the procedure, a potential barrier to following the established procedures [7]. A study of the effectiveness of implementing a central venous catheter care bundle showed that provision of adequate equipment at the point of need was essential in supporting frontline staff in complying with evidence-based care bundle guidelines [8]. Another study demonstrated the need for a standardised list of equipment for a catheter insertion kit that included all supplies required to adhere to recommended guidelines [9]. However, no studies have examined equipment availability for the more frequently undertaken insertion of peripheral intravenous cannulae.

Our objectives were to measure the reliability of equipment availability for the insertion of peripheral intravenous cannulae in a selection of clinical areas in three UK hospital organisations, to identify differences between organisations, to explore the causes of poor reliability, and to suggest areas for improvement.

## Methods

The study was descriptive and employed a mixed methods approach. An existing UK care bundle relating to the insertion of peripheral intravenous cannulae [10] was used to identify a core list of equipment required. We then measured the reliability of the system to deliver these pieces of equipment when needed for peripheral intravenous catheter insertions, and conducted interviews with key staff to explore how the supply chains worked and the factors perceived as contributing to equipment unavailability.

### Setting

The study was conducted in three English hospital organisations (A,D and F). Organisations A and D served large metropolis areas while organisation F was located in a suburban area. All organisations were subject to the Department of Health guidance [10]. Table 1 summarises the clinical areas studied. Data were collected in summer 2009. None of the three organisations changed any relevant procedures during the course of the study.

**Table 1:** Areas studied and staff involved in cannulation, at each participating organisation

**Site**	**Cannulation performed by**	**Departments studied**	**Cannulation supplies used**
A	Nurses, doctors and medical assistants.	Accident and emergency department and two acute admission wards.	Cannulation packs used which included one cannula per pack. Disposable tourniquet not included in pack.
D	Doctors and advanced clinical practitioners.	Accident and emergency department.	Cannulation packs used which do not include a cannula. Cannula selected separately. Disposable tourniquet not included in pack.
F	Medical assistants.	Medical wards.	Cannulation packs were not used and all materials selected separately.

### Definitions

Reliability was defined as the percentage of all peripheral catheter insertions for which all items of equipment were available, and suitable for use. The equipment deemed necessary comprised the following items from the care bundle [10]:

 · Hand hygiene facilities (hand washing facilities or alcohol gel)

 · Personal protection (usually gloves alone but when indicated may include apron, goggles etc.)

 · Skin preparation (e.g. 2% chlorhexidine in 70% isopropylalcohol)

 · Peripheral venous cannula of appropriate size/gauge

 · Specific peripheral intravenous cannula dressing

We also added a suitable clean tourniquet and a sharps disposal bin to the list of equipment required, following consultation, pilot work, and comparison with the equivalent central venous catheter care bundle [11].

An equipment failure was defined as one of the above items not being present, or not being suitable for use, at the time it was needed.

### Data collection

#### Reliability of equipment availability

Data were collected over a period of four weeks, including weekends, in each organisation. A data collection form was designed to measure the prevalence of equipment failures during catheter insertion, together with the perceived impact on patient safety of each equipment failure. The forms were distributed to all participating clinical areas, and staff performing cannulations were asked to complete a form after each procedure. Site D modified the data collection form slightly for local use, based on a local care bundle audit, so that the data could be used for both purposes.

#### Exploring the systems failures involved

We aimed to interview 5 members of staff in each organisation, purposively sampling to obtain the views of nurses, doctors, medical assistants and managers. Potential participants were approached by the researcher through local contacts in each organisation. Interviews were conducted face to face using a semi-structured interview schedule developed as part of a larger study of reliability failures [12], were taped and then transcribed. Interviewees gave written informed consent. Staff were asked similar questions, accepting that some may only know some components of the supply chain and cannulation process. An established coding frame [13] was adapted by one researcher and then revised by a second. Coding of 50% of the interviews was checked by a second researcher; any differences in coding were discussed and agreed between the two.

The study had NHS ethics approval. Interviewees gave written informed consent, and were given the opportunity to review their transcripts before analysis.

## Results

### Reliability of equipment availability

Across the study organisations a total of 350 peripheral venous cannulae were studied. A total of 47 incidents of non-availability or non-functional equipment occurred in 46 cannulation operations (one cannulation procedure was associated with two equipment problems), representing a prevalence of 13.1%. The cannulation process therefore had a reliability of 86.9%. Non-availability of a suitable sharps bin accounted for a high proportion (n = 25; 53% of all incidents). Of the 46 cannulations with equipment problems, 44 (96%) were perceived by participants to have either no or minimal impact on patient safety.

### Variability between organisations

At organisation A, 76 cannulae were inserted and 15 problems identified (19.7%). All incidents related to unavailability of cannulae, tourniquets and sharps bins. In the two incidents involving cannulae, peripheral cannula packs with the appropriate cannulae size were not available. In five cases, staff stated that the small sharps bins provided were unsafe to use. Tourniquets were not available in eight cases and staff used disposable gloves as tourniquets instead.

At organisation D, 62 cannulae insertions were studied with seven (11.3%) experiencing equipment problems. At this organisation the problems were related to availability of skin preparation and correct dressings; this arose where peripheral cannula packs were out of stock and staff had to collect items separately before proceeding with cannulation.

At organisation F, 212 cannulae insertions were studied, with 24 (11.3%) experiencing 25 equipment problems. This organisation experienced problems with availability of cannulae, sharps bins, skin preparation and dressings.

None of the sites reported equipment problems in relation to hand hygiene and personal protection. No difference was found between sites in relation to the prevalence of equipment problems (p = 0.2; chi square test). The types of equipment problem are summarised in Table 2.

**Table 2:** Non-availability of equipment in each organisation

**Item**	**Organisation A(% of cannulations)**	**Organisation D(% of cannulations)**	**Organisation F(% of cannulations)**	**TOTAL(% of** **cannulations)**
Total cannulations	76	62	212	350
Hand hygiene facilities	0	0	0	0
Personal protection e.g. gloves	0	0	0	0
Skin preparation e.g. 2% chlorhexidine	0	2(3.2%)	1(0.5%)	3(0.9%)
Clean tourniquet	8(11%)	0	0	8(2.3%)
Intravenous cannula	2(3%)	0	2(1.0%)	4(1.1%)
Specific intravenous cannula dressing	0	5(8.0%)	2(1.0%)	7(2%)
Sharps disposal bin	5(7%)	0	20(9.4%)	25(7.1%)
TOTAL FAILURES	15(19.7%)	7(11.3%)	25(11.8%)	47(13.4%)
RELIABILITY	80.3%	88.7%	88.2%	86.9%

### Systems failures analysis

A total of eight interviews were conducted across the three organisations (a nurse, a doctor and a ward manager at organisation A, two doctors and a nurse in organisation D, and a medical assistant and a ward manager at organisation F). Factors perceived to have contributed to the non-availability of the right equipment for the safe insertion of peripheral venous cannulae were grouped under the categories of work environment, team work, task factors and individual factors. Quotes are presented in Table 3.

**Table 3:** Illustrative quotes

**No. **	**Details**
1.	*So again in terms of familiarity and ease of usage then it’s going to be difficult for the staff (participant 2, site A)*
2.	*And some store people who stock up rooms will put stuff where they think it needs to go and a nurse may very well put it completely somewhere else….all the wards have different layouts (participant 2, site F)*
3.	*They put it [the order] on a Monday and we’re expecting it on a Thursday and on Thursday it doesn’t come. … then we’ll find out that you know it’s out of stock or they have given us different stuff (participant 2, site A)*
4.	*Mostly Bank Holidays, mostly when the holidays are coming, mostly and sometimes it’s Saturdays. It really depends sometimes, like it’s mostly Bank Holidays (participant 1, site A)*
5.	*We’ve got trolleys set up for peripheral insertion, the cannulae and syringes, everything, we do have a problem with it being stocked (participant 2, site A)*
6.	*Minors, there’s problems with the equipment in minors, I think because there’s less insertion of IV lines put down in there, that stock gets depleted and not replenished (participant 2, site A)*
7.	*It might be their job and yet they’re not the ones that use it so … that you may not, that wouldn’t be your priority because it’s not something that’s on your radar (participant 1, site F)*

#### Work environment factors

Purchasing policies meant that brands and types of equipment were perceived to often change, and staff were not always familiar with new equipment. Store rooms were also perceived to be user-unfriendly (quotes 1 and 2).

#### Teamwork factors

Breakdown of communication between teams during the ordering and supply processes may have affected the availability of equipment (quotes 3 and 4).

#### Task factors

The clarity and structure of certain tasks may have lead to misunderstanding and confusion among staff and affect activities, particularly where cannulation was less commonly performed (quotes 5 and 6).

#### Individual factors

Gaps in staff knowledge were perceived to influence the efficient execution of their job and the provision of equipment (quote 7).

## Discussion

### Key findings

Establishing care bundles is not enough to support patient safety; suitable systems are also needed to support these care bundles and to ensure that the required equipment is available at the point of need. The reliability with which appropriate equipment was available for peripheral intravenous cannulation, as defined by the cannulation care bundle [10] plus additional requirements for tourniquets and sharps bins, was low. Levels of reliability were similar (80.3%; 88.7% and 88.2%) across three study organisations, but the types of failure were highly variable. In organisation A the main issues were lack of a clean tourniquet and sharps bins; in organisation D, it was availability of dressings and in F, it was sharps bins. This indicates that the problems are not insurmountable as at least one organisation had no failures in each category.

We found that the supply chain set up to deliver the correct equipment for peripheral venous cannulation in our study sites was based on old-style routine reordering systems. There were no apparent feedback loops to ensure replenishment of stocks.

The resulting harm due to these equipment problems is unknown, although staff perceived it to be low. The availability of an empty sharps bin and the correct size of cannula appear to be common issues.

This paper is therefore the first to present details of where failures are occurring and potential areas where improvement could be focussed. In particular, the work highlights the many human factors related to the supply chain in intravenous cannulation.

### Interpretation

Development of supply chain systems to ensure adequate stock control and availability and replenishment of full sharps bins may improve the reliability of availability of equipment. The need for extra, or different, cannulae during a procedure should also be taken into account when designing cannulation packs. Communication along the supply chain was a key issue. Interviews highlighted the requirement for those responsible for ordering, supply and restocking to be aware of the needs of frontline staff. Staffing issues can cause problems with restocking, particularly if restocking is seen as a particular person’s responsibility (or no-one’s). The lack of sharps disposal bins often resulted in staff taking their sharps to another location for disposal increasing the risk of needlestick injuries to themselves. It may also be that staff familiarity with the type of equipment used has an impact on patient safety, in which case it is important that this be factored into the equipment that is made available. The use of equipment packs will be influenced by their design and way in which they are stored on the ward.

### Strengths and limitations

The strengths of our study are that we used a nationally-accepted care bundle and so results represent reliability against agreed standards, with the additional requirement for a clean tourniquet and sharps bin. We also used the same definitions and methods in each of three organisations, thus facilitating comparison. In relation to limitations, equipment availability was measured using self-reporting which may be open to bias, although we believe that this was minimised by anonymity and clear explanation of the purpose of the study, focusing on systems rather than individuals. The assessment of the impact on patient safety was based on the subjective opinion of the staff involved. Adverse events are likely to be rare, and remote from the original cannulation, and so staff may not be able to accurately predict the risks. We also assessed risks from the patient’s perspective and so will not have captured the risks to staff resulting from unavailability of sharps bins, resulting in staff transporting used sharps around their clinical area. There were some differences between organisations in the clinical areas studied. Interviews were few in number, due to the limited time frame available to complete all interviews on all sites; demands on staff meant that interviews often got cancelled at short notice. Finally, generalisability to other hospitals, and in particular to other countries, is unknown.

### Recommendations

It appears that the majority of issues would be resolved if:

 Standardised packs were used, but available with a variety of cannula sizes, as well as back-up supplies.

 The use of disposable tourniquets may increase reliability if incorporated into the packs; there is also evidence that reusable tourniquets are associated with the risk of infection [14,15].

 Systems should be developed to ensure reliable restocking, including systems to inform those responsible for restocking supplies when supplies are low.

 Equipment is stored as locally as possible to the location where cannulation is being undertaken.

 Effective team work and communication is needed to ensure restocking when replenishment is required, and so that those responsible for restocking are aware of the consequences of the stock not being available, or of sharps bins not being available.

A basic pack of equipment in a trolley which also includes spares, more variable equipment and sharps disposal facilities, which is taken to the patient and then returned to a central restocking point may resolve many of these issues. Such solutions should include consultation with users [16].

More in-depth study would be required across multiple organisations to determine whether the failure modes in the study organisations are representative of the wider NHS. The Department of Health audit tool for measuring staff compliance with peripheral cannulation care bundle may not detect work-arounds which have the potential to increase risk as it examines whether or not actions are completed. It may be improved by a simple root cause analysis to determine whether equipment non-availability was a contributory factor to actions not being completed, and how availability could be improved. It is likely that availability of sharps bins may be a significant problem and organisations should look at how to make these readily available at the point of care.

## Conclusions

Reliability of supply of correct functional equipment for cannulation was about 87%. In 13% of cannulations, staff therefore had to work without the right equipment being available when needed. No single issue was seen across all organisations, suggesting that each organisation has resolved some issues. Sharps bin supplies close to the place of cannulation and availability of clean tourniquets were important issues. It may be possible to improve the reliability of the availability of equipment for peripheral venous cannulation by using supply chain management principles. For each organisation, there are also some issues that could be resolved by learning from other organisations in the study. Future research could look at systems level interventions and their impact.

## Abbreviations

NHS: National Health Service; UK: United Kingdom; US: United States.

## Competing interests

The authors declare that they have no competing interests

## Authors’ contributions

BDF and CV conceived the study and participated in design, co-ordination and analysis. VD, MC and AH designed the methods of data collection. VD co-ordinated data collection, conducted interviews and performed data analysis. BDF led the writing of the report and paper. All authors contributed to paper-writing, and have read and approved the final manuscript.
